# Functional Annotation of Proteomic Data from Chicken Heterophils and Macrophages Induced by Carbon Nanotube Exposure

**DOI:** 10.3390/ijms15058372

**Published:** 2014-05-12

**Authors:** Yun-Ze Li, Chung-Shi Cheng, Chao-Jung Chen, Zi-Lin Li, Yao-Tung Lin, Shuen-Ei Chen, San-Yuan Huang

**Affiliations:** 1 Department of Animal Science, National Chung Hsing University, Taichung 40227, Taiwan; E-Mails: joezxcvb2000@yahoo.com.tw (Y.-Z.L.); terry80149@hotmail.com (C.-S.C.); zl3350103@yahoo.com.tw (Z.-L.L.); 2 Proteomics Core Laboratory, Department of Medical Research, China Medical University Hospital, Taichung 40402, Taiwan; E-Mail: cjchen@mail.cmu.edu.tw; 3 Graduate Institute of Integrated Medicine, China Medical University, Taichung 40402, Taiwan; 4 Department of Soil and Environmental Sciences, National Chung Hsing University, Taichung 40227, Taiwan; E-Mail: yaotung@nchu.edu.tw; 5 Center of Nanoscience and Nanotechnology, National Chung Hsing University, Taichung 40227, Taiwan; 6 Agricultural Biotechnology Center, National Chung Hsing University, Taichung 40227, Taiwan; 7 Center for the Integrative and Evolutionary Galliformes Genomics, iEGG Center, National Chung Hsing University, Taichung 40227, Taiwan

**Keywords:** carbon nanotube, macrophage, heterophil, protein expression, cell migration, chicken

## Abstract

With the expanding applications of carbon nanotubes (CNT) in biomedicine and agriculture, questions about the toxicity and biocompatibility of CNT in humans and domestic animals are becoming matters of serious concern. This study used proteomic methods to profile gene expression in chicken macrophages and heterophils in response to CNT exposure. Two-dimensional gel electrophoresis identified 12 proteins in macrophages and 15 in heterophils, with differential expression patterns in response to CNT co-incubation (0, 1, 10, and 100 μg/mL of CNT for 6 h) (*p* < 0.05). Gene ontology analysis showed that most of the differentially expressed proteins are associated with protein interactions, cellular metabolic processes, and cell mobility, suggesting activation of innate immune functions. Western blot analysis with heat shock protein 70, high mobility group protein, and peptidylprolyl isomerase A confirmed the alterations of the profiled proteins. The functional annotations were further confirmed by effective cell migration, promoted interleukin-1β secretion, and more cell death in both macrophages and heterophils exposed to CNT (*p* < 0.05). In conclusion, results of this study suggest that CNT exposure affects protein expression, leading to activation of macrophages and heterophils, resulting in altered cytoskeleton remodeling, cell migration, and cytokine production, and thereby mediates tissue immune responses.

## 1. Introduction

The physiochemical properties of most materials can be altered by reducing their size to nanoscale; such nanomaterials enable many revolutionary applications [1]. Nanomaterials are commonly found in electronics, rubber tires, sporting equipment, foods, preservatives, and pharmaceuticals, items which are intimately part of our daily life [2]. However, due to their small size, they have large surfaces for contacting a target, allowing greater mantle, adsorption and interaction. As the applications of nanomaterials are increasingly widespread, they can easily be taken up by the respiratory tract, gastrointestinal tract, and even the skin of humans or animals, creating concern about their potential health risks.

Carbon nanotube (CNT) is an allotrope of carbon with a cylindrical nanostructure, the diameter ranging from 3 to 40 nm and approximately 1 μm in length. Because of its unique length-to-diameter ratio, it is ultralight and possesses high mechanical strength, electrical conductivity, thermal conductivity, metallic or semimetallic behavior and a high surface area, thus CNT is considered one of the most promising manufactured nanomaterials [3]. For example, its use has been proposed for sentinel lymph node tracers [4], drug delivery [5], and for high temperature superconductive materials [6]. On the other hand, due to its fibrous shape, extreme aspect ratio, low specific density and low solubility, CNT could exhibit toxicity similar to that of asbestos [7].

Over the last few years, many studies have reported that CNT can cause inflammation in the lungs of rodents, as well as formation of granulomas and/or fibrotic responses [8–10]. Furthermore, genotoxic and apoptotic effects of CNT in lung epithelial cells and/or immunocytes have also been reported [8,11–14].

Previous studies have shown that, in the reticuloendothelial system, macrophages may engulf CNT, but it is difficult for them to metabolize [15], and has greater cytotoxicity than quartz and fullerene [16]. Heterophils are highly phagocytic, polymorphonucleated white blood cells, resembling mammalian neutrophils, and are the second most numerous immunocytes in the circulation of avian species [17]. Heterophils function to engulf foreign substances and respond to various chemotactic stimuli, particularly invading microorganisms. They are the first arrival immune cells infiltrating into the damaged parts. Previous studies on the toxicity, DNA damage and pathogenic effects of CNT have mostly focused on mammals. Evaluating the effects of CNT exposure on chicken macrophages and heterophils can provide information about how CNT affects tissue immune response and pathogenesis in domestic fowl.

Applied to environmental toxicology, proteomics is an important tool for evaluating the growing environmental threat posed by nanoparticles and endocrine disrupting agents [18]. Therefore, this study first profiled gene expression in chicken macrophages and heterophils exposed to CNT using a proteomic approach, and then analyzed their differential expression in functional annotations. Finally, to confirm the annotated functions through gene ontology analysis, we analyzed cell migration and viability, as well as cytokine secretion.

## 2. Results and Discussion

### 2.1. Cytotoxicity of Carbon Nanotubes on Chicken Macrophages and Heterophils

**Figure 1.**
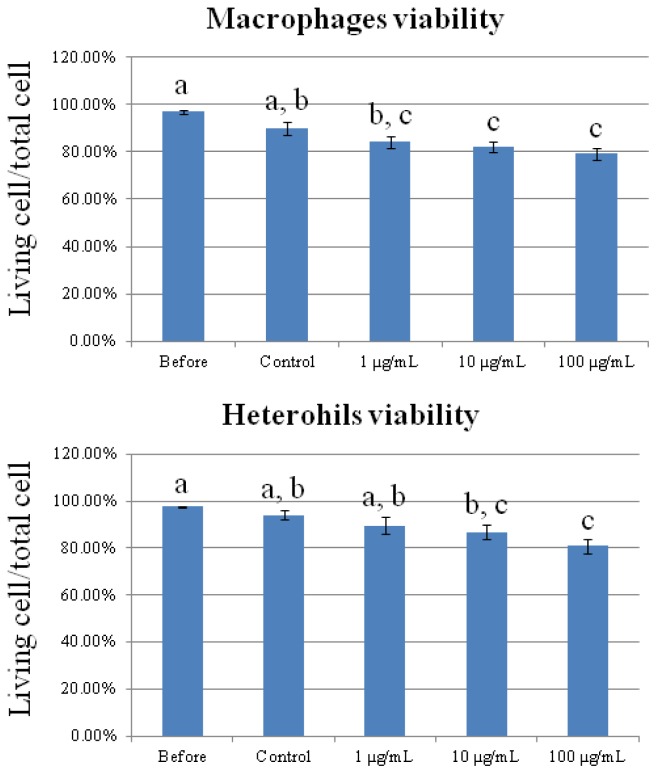
Viability of chicken macrophages and heterophils after 6 h of exposure to carbon nanotubes. Values are the mean ± SE of three batches. ^a–c^ The means with different superscripts differ significantly (*p* < 0.05).

We performed cell viability assays to evaluate the cytotoxicity of CNT on chicken macrophages and heterophils. Figure 1 shows a dose-dependent suppression of cell viability upon exposure to CNT. The viability of macrophages and heterophils decreased significantly in the 10 and 100 μg/mL CNT-treated groups (*p* < 0.05). The result indicated that the toxic doses to macrophages and heterophils were higher than 10 and 100 μg/mL, respectively.

### 2.2. The Effect of Carbon Nanotubes on Macrophage Protein Expression

**Figure 2.**
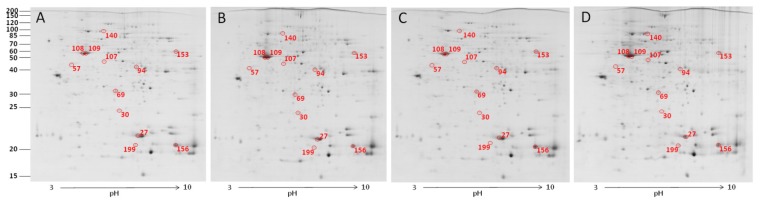
Protein profiles and differentially expressed spots of CNT (Carbon nanotube)-treated chicken macrophages. Macrophages were treated with 0 μg/mL (**A**); 1 μg/mL (**B**); 10 μg/mL (**C**); and 100 μg/mL (**D**) carbon nanotubes, then proteins were extracted for 2-DE analysis. Spot numbers refer to the numbers in Table 1.

**Figure 3.**
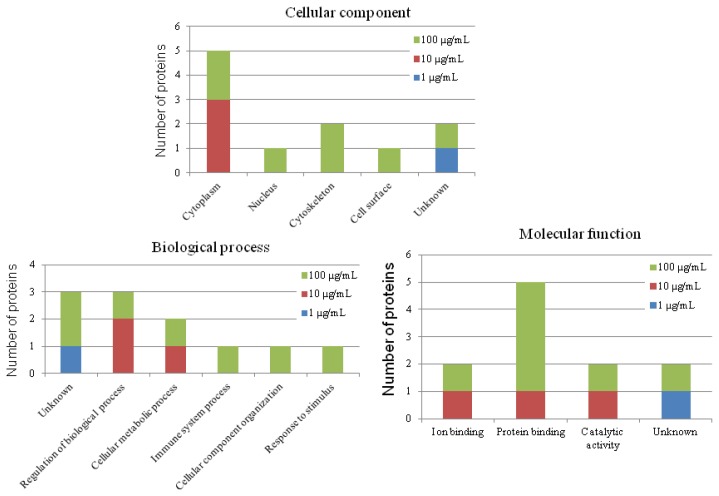
Gene ontology (GO) annotation of differentially expressed proteins in carbon nanotube-treated chicken macrophages. The original GO annotations were downloaded from the NCBI Entrez Gene database (Bethesda, MD, USA). The percentages are the total hits, divided by the number of annotated proteins for the category.

To profile protein expression by CNT exposure, 2-DE analysis was performed. Figure 2 illustrates the protein profiles of chicken macrophages in response to various levels of CNT. In total, 12 of 202 quantified spots differed significantly among treatments (*p* < 0.05). Of the 12 protein spots, five were upregulated and four were downregulated in the CNT-treated groups (Table 1). The differentially expressed proteins were identified by MALDI-TOF (Matrix Assisted Laser Desorption/Ionization Time-of-Flight) and MALDI TOF/TOF MS (Mass Spectrometry); the detailed identifying information is listed in Supplementary Table S1. Ten of them were successfully identified. Among these differentially expressed proteins, similar to hepatoma-derived growth factor (high-mobility group protein 1-like), high mobility group protein HMG1, similar to Pdlim1 protein isoform 1, adenosine deaminase, cytoplasmic actin type 5, beta-actin, heat shock protein 70, and phosphoglycerate kinase were found significantly changed after toxic dose treatment. To further characterize the differentially expressed proteins, the proteins with known identities were classified according to their GO (gene ontology) annotations. Figure 3 demonstrates that most of the differentially expressed proteins were located in the cytoplasm (50%) and involved in the molecular function of protein interaction (33%). In the biological process annotation, the differentially expressed proteins primarily participated in regulating biological processes (25%), cellular metabolic processes (17%), organization of cellular components (17%), and responding to stimuli (17%). The known functions of the identified proteins are related to cytoskeleton organization, cellular energy metabolism, and immune response (Supplementary Information, Table S1).

### 2.3. The Effect of Carbon Nanotubes on the Protein Expression of Heterophils

**Figure 4.**
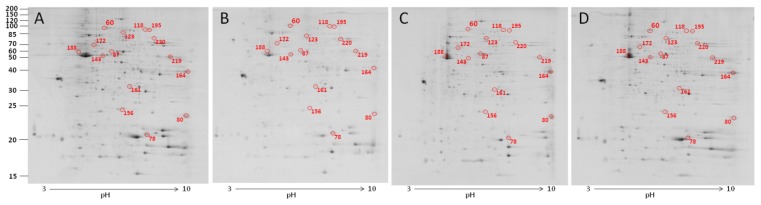
Protein profiles and differentially expressed spots of CNT-treated chicken heterophils. Heterophils were treated with 0 μg/mL (**A**); 1 μg/mL (**B**); 10 μg/mL (**C**); and 100 μg/mL (**D**) carbon nanotubes, then proteins were extracted for 2-DE analysis. Spot numbers refer to the numbers in Table 2.

**Figure 5.**
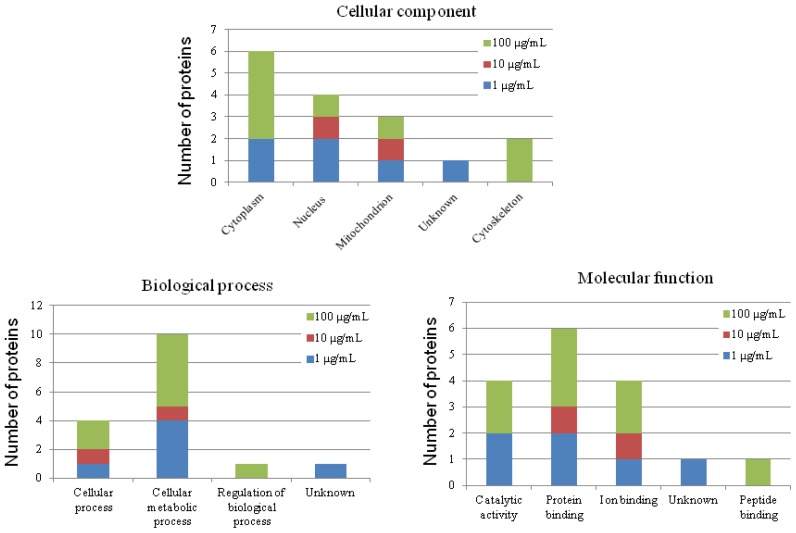
Gene ontology (GO) annotation of differentially expressed proteins in CNT-treated chicken heterophils. The original GO annotations were downloaded from the NCBI Entrez Gene database. The percentages are the total hits, divided by the number of annotated proteins for the category.

In heterophils, 2-DE analysis suggested that 15 out of 229 protein spots differed significantly in response to CNT exposure (*p* < 0.05; Figure 4). Eight of them were upregulated and four were downregulated (Table 2). The detailed identities of the differentially expressed protein spots are listed in Supplementary information, Table S2. We successfully identified 14 of the differentially expressed protein spots (Supplementary information, Table S2). The significantly differentially expressed proteins after toxic dose treatment included gelsolin precursor, peptidylprolyl isomerase A, moesin-like, phosphoglycerate mutase 1, phosphoglycerate kinase, and similar to transketolase. GO annotation revealed that most of the differentially expressed proteins were located in cytoplasm (47%), and related to the molecular function of protein interaction (40%) (Figure 5). As for biological process, most of the differentially expressed proteins participated in cellular metabolic processes (53%). The known functions of the identified proteins in CNT-treated heterophils are related to cytoskeleton organization and cellular energy metabolism (Supplementary information, Table S2).

### 2.4. Validation of Protein Expression by Western Blot

**Figure 6.**
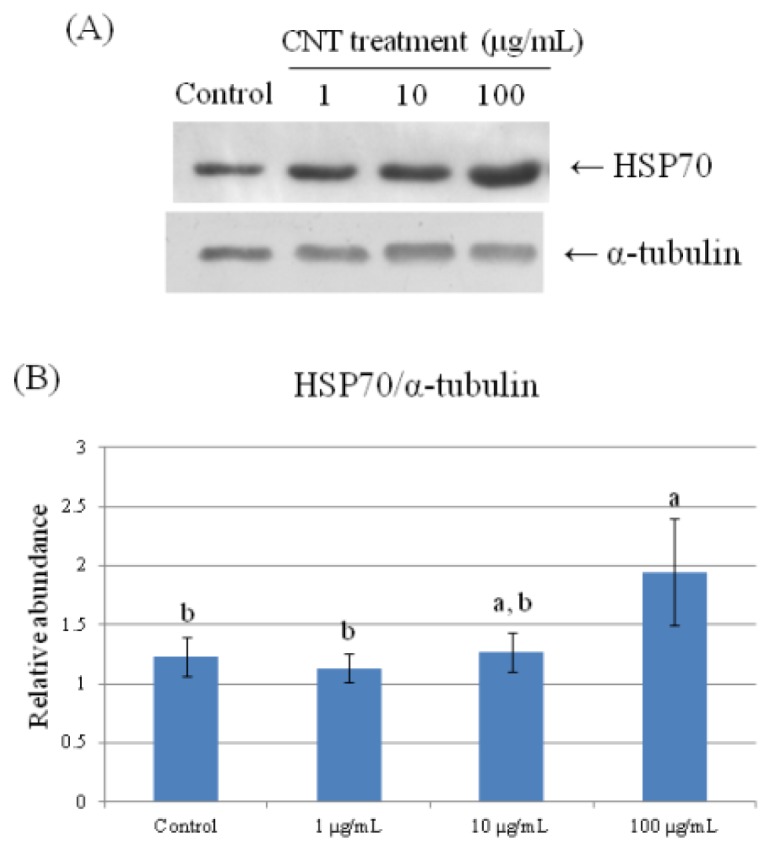
Validation of HSP70 in CNT-treated chicken macrophages by Western Blot analysis. Samples were subjected to 12.5% SDS-PAGE. The protein in the polyacrylamide gel was transferred to a nitrocellulose membrane. (**A**) The immunoblot was produced by incubating the membrane with antibodies against HSP70 and α-tubulin; (**B**) The relative abundance of HSP70/α-tubulin was analyzed with Total Lab^®^ (UltraLum, Claremont, CA, USA). Values are the mean ± SE of three batches. ^a,b^ The means with different superscripts differ significantly (*p* < 0.05).

**Figure 7.**
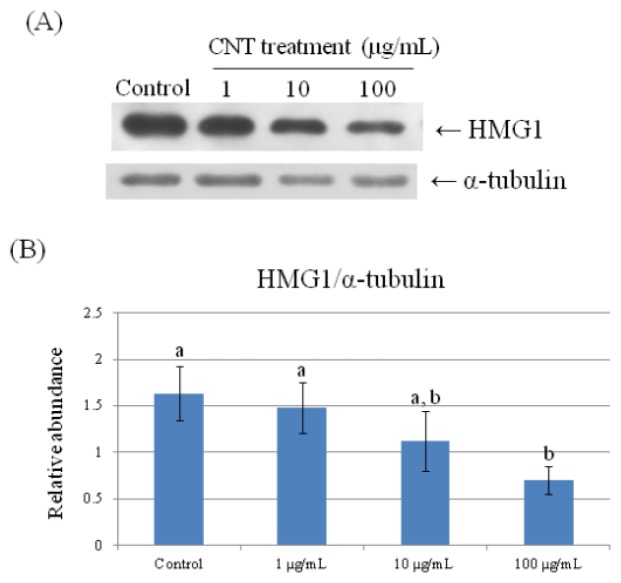
Validation of HMG1 in CNT-treated chicken macrophages by Western Blot analysis. Samples were subjected to 12.5% SDS-PAGE. The protein in the polyacrylamide gel was transferred to a nitrocellulose membrane. (**A**) The immunoblot was produced by incubating the membrane with antibodies against HMG1 and α-tubulin; (**B**) The relative abundance of HMG1/α-tubulin was analyzed with Total Lab^®^. Values are the mean ± SE of three batches. ^a,b^ The means with different superscripts differ significantly (*p* < 0.05).

**Figure 8.**
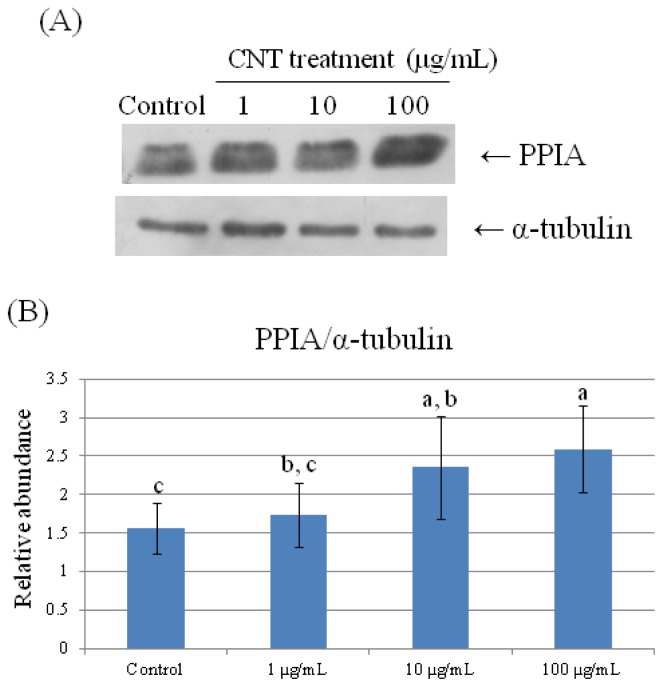
Validation of PPIA in CNT-treated chicken heterophils by Western Blot analysis. Samples were subjected to 12.5% SDS-PAGE. The protein in the polyacrylamide gel was transferred to a nitrocellulose membrane. (**A**) The immunoblot was produced by incubating the membrane with antibodies against PPIA and α-tubulin; (**B**) The relative abundance of PPIA/α-tubulin was analyzed with Total Lab^®^. Values are the mean ± SE of three batches. ^a–c^ The means with different superscripts differ significantly (*p* < 0.05).

To further validate the results of the 2-DE analysis, we used Western Blot analyses to confirm HMG1, HSP70, and PPIA expression (Figures 6–8). Consistent with the results of the 2-DE analysis, we found that CNT-induced HMG1, HSP70, and PPIA expression changed in a dose-dependent manner (*p* < 0.05).

### 2.5. Cell Migration Assay

**Figure 9.**
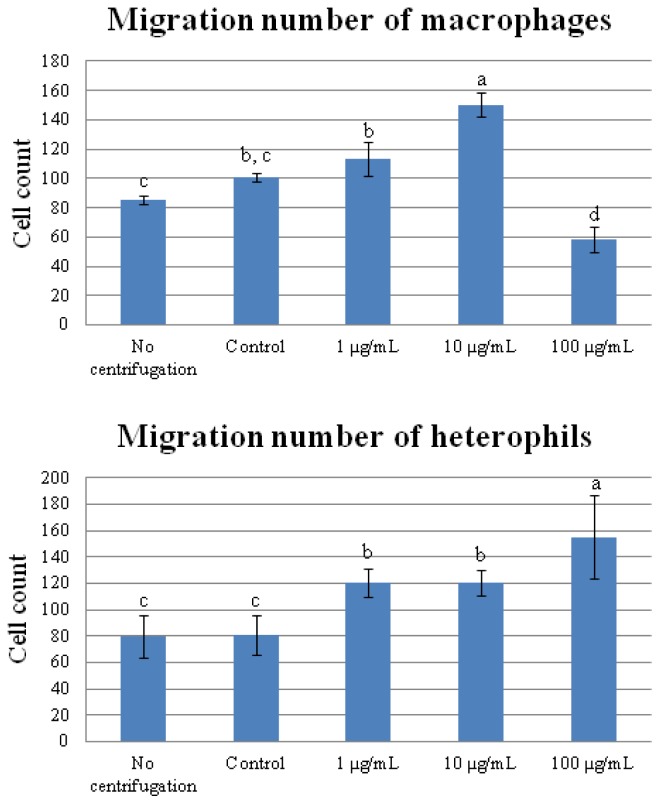
Carbon nanotube treatments enhance migration of chicken macrophages and heterophils. The chicken macrophages and heterophils were added to the upper chambers of inserts containing 5.0 μm pore membranes and incubated at 37 °C for 40 min. Values are the mean ± SE of three batches. ^a–c^ The means with different superscripts differ significantly (*p* < 0.05).

To confirm the GO annotation, we used cell migration analysis to examine the functions altered by CNT exposure. To prevent the CNT from interfering with the observation, the cells were centrifuged to remove CNT before the migration assay. Results showed that centrifugation did not affect the cell migration in either macrophages or heterophils (Figure 9). The cell migration rate of macrophages increased significantly (*p* < 0.05) in the 10 μg/mL CNT group, but decreased in the 100 μg/mL CNT-treated group (*p* < 0.05). In contrast, the migration of heterophils increased significantly with increasing amounts of CNT (*p* < 0.05).

### 2.6. IL-1β Secretion

**Figure 10.**
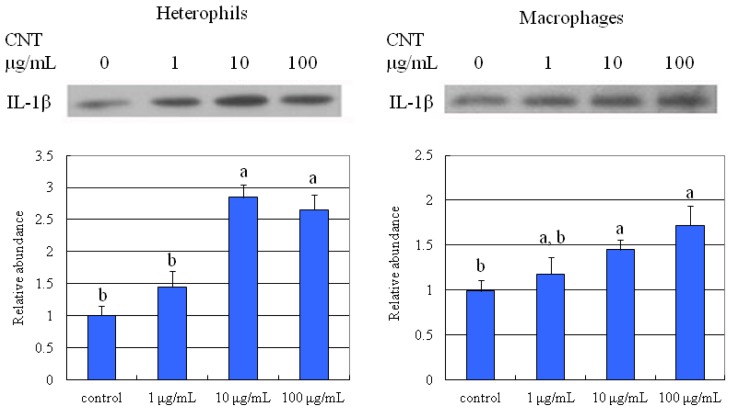
Carbon nanotube treatments promoted IL-1β production of chicken macrophages and heterophils. Samples were subjected to 12.5% SDS-PAGE. The protein in the polyacrylamide gel was transferred to a nitrocellulose membrane. (**A**) The immunoblot was produced by incubating the membrane with antibodies against IL-1β; (**B**) The abundance of IL-1β was analyzed with Total Lab^®^. Values are the mean ± SE of three batches. ^a,b^ The means with different superscripts differ significantly (*p* < 0.05).

We then used cytokine secretion to examine the mediation of immune cells in the development of possible inflammatory response in damaged tissues. At higher levels (10 and 100 μg/mL), CNT exposure significantly promoted IL-1β production in both chicken heterophils and macrophages (Figure 10).

### 2.7. Putative Roles of the Differentially Expressed Proteins in the Functions of Chicken Macrophages and Heterophils

Carbon nanotubes are promising materials with diverse applications in different fields of research [19]. However, the structure of CNT is similar to that of asbestos fibers [7] (Maynard *et al.*, 2004), leading to serious concerns about its biosafety for human or animal health. Results of this study showed a dose-dependent suppression of cell viability in CNT-treated macrophages and heterophils (Figure 1). Previous studies have shown that CNT treatment can induce oxidative stress in human keratinocytes [20], A549 cells [21], kidney epithelial cells [13], and skin epithelial cells [14]. These reactive oxygen species (ROS)-mediated pathways involved in cell death are related to NF-κB signaling, AP-1 [22], and caspase-3/7 activation [23]. The trigger causing generation of ROS has been attributed to the residual metal catalysts used during CNT synthesis, possibly via the Fenton reaction [24]. He *et al.* [25] observed that CNT induced oxidative stress and cytochrome c release, causing cell death through a common mechanism of mitochondrial damage.

Migration and infiltration of immunocytes into the sites that become inflamed are an essential aspect of the immune response in damaged tissues. Macrophages and leucocytes are attracted to the site of inflammation by cytokines and chemokines that can go on to induce cytoskeleton remodeling and activate cytoskeleton-associated proteins to enhance infiltration ability [26]. In this study, the expression levels of 12 proteins differed significantly among the 202 quantified protein spots in chicken macrophages after exposure to CNT (Table 1). Some of these proteins have been shown to relate to cell migration and immune response. Among the CNT-treated heterophils, 15 out of 229 protein spots differed significantly (Table 2). Most of the proteins are classed as cellular proteins and participate in cell migration.

HSP70 acts as a chaperone in maintaining normal protein folding and thus protects cells from stress insults [27]. HSP70 released from nectrotic cells or exogenous HSP70 treatment has been shown to induce TNF-α, IL-12, and IL-1β secretion via NF-κB pathway in macrophages, activating antigen presenting cells [28,29]. Thus, CNT-induced upregulation of HSP70 and IL-1β secretion in macrophages (Table 1, Figure 6) suggests that CNT exposure results in activating stress-related signals.

Adenosine deaminase (ADA) is highly expressed at inflamed sites and its activity is required for inflammatory response [30]. In some infectious diseases, the plasma level of ADA rises, representing a compensatory mechanism to increase the release of inflammatory mediators [31]. Our results thus confirmed the increase of ADA expression and IL-1β release caused by CNT exposure and demonstrate the importance of cytokines released from macrophages in mediating inflammatory response (Table 1).

High mobility group protein (HMG1) and heat shock protein (HSP) are likely to act as the alerting signal in general inflammation and sterile inflammation [32]. In macrophages, monocytes, and dendritic cells, HMG1 can also act as a cytokine interacting with toll like receptor (TLR) 4 to mediate secretion of other cytokines and NF-κB expression, and subsequently ROS release via TLR-dependent activation of NADPH oxidase [33,34]. HMG1 has a strong affinity with advanced glycation end products (AGEs) produced by injured tissues; blocking the interaction of HMG1 with AGEs has been shown to dramatically suppress the chemotactic ability of macrophages [35].

Differential expression of actin type 5, β-actin, and PDLIM1 (similar to Pdlim1 protein isoform 1) play important roles in cell migration. Actin type 5 (gamma-actin) is essential for organizing meshwork in cortical and lamellipodial structures during cell movement, and β-actin is preferentially localized in stress fibers and cell-cell contacts. Both actin type 5 and β-actin are closely associated with cell motility [36]. PDLIM1 is the enigma protein containing PDZ, ZN, and LIM domains and thus acts to connect actin stress fibers and kinases, mediating signal transduction to regulate cell morphology and motility [37]. In this study, we observed that lower levels of CNT exposure promoted macrophage motility but a higher level of CNT exposure exerted a repressive effect on cell migration. The functional results of macrophage motility are coherently matched the lowest expression levels of PDLIM1 and HMG1 and the migration rate in 100 μg/mL CNT-treated macrophages (Table 1, Figure 9). The mechanism needs further study.

Gelsolin is a cytoskeleton-regulated protein involved in actin remodeling, morphology maintenance, cell growth and division [38]. Tanaka *et al.* [39] reported that gelsolin expression decreases in the early stages of malignant transformation, and an increase of gelsolin expression probably plays a critical role in converting a superficial tumor to an invasive tumor. In addition, gelsolin gene knockout mice showed less acute injury with fewer infiltrating neutrophils in the lungs [40]. The REM (ezrin, radixin, moesin) protein provides a linkage between plasma membranes and actin cytoskeletons. They also participate in diverse functions like endocytosis, exocytosis, adhesion, and migration [41]. Boldt *et al.* [42] also found that the FPRL-1 signaling target proteins such as l-plastin, moesin, cofilin, and stathmin can regulate the motility of polymorphonuclear neutrophils. In moesin deficient mice, infiltration of neutrophils was reduced three-fold in lipopolysaccharide’-induced skin lesions [43]. In this study, gelsolin and moesin expression increased with CNT concentration (Table 2), concomitantly with increased migration rate of heterophils (Figure 9). Our results suggest that CNT exposure activates heterophils, the first immune cells to arrive at the site of inflammation.

Foldases, such as protein disulfide isomerase and peptidyl-prolyl-cis-trans isomerase (PPIase), function to facilitate protein folding. Peptidylprolyl isomerase A (PPIA), also called cyclophilin A, belongs to the PPIase family [44,45]. Although PPIA is an intracellular protein, it also acts as a pro-inflammatory product released from activated macrophages and inflamed tissues to induce chemotaxis [46]. Since blocking of extracellular PPIA has been shown to reduce tissue neutrophils up to 50% [47], upregulation of PPIA by CNT exposure in heterophils (Table 2, Figure 8) suggests a chemotactic effect elicited by CNT.

Macrophages and heterophils are both phagocytes in the innate immune defense system. However, the expression levels of HMG1 and PPIA are opposite in the two cell types (Tables 1 and 2). CNT-induced expression of HMG1, ADA, and HSP70 changed significantly in macrophages, but not in heterophils. All of these gene products are related to cytokine secretion, implying that macrophages may mediate immune responses primarily by recruiting other immunocytes to inflamed sites [48]. In contrast, proteins associated with cell migration (including gelsolin, moesin, beta-actin, and PPIA) and metabolism, including aconitate hydratase, and phosphoglycerate mutase 1 (PGAM1), are upregulated in CNT-treated heterophils (Table 2). The results confirm the notion that heterophils migrate faster than macrophages to inflamed sites for innate immunity, such as phagocytosis [49]. In addition to the role of innate immunity, activation of macrophages as antigen presenting cells also functions to mediate adaptive immunity through cytokine and chemokine production for recruiting other immunocytes to the site of inflammation [48].

### 2.8. Possible Mechanisms of CNT-Induced Cell Death and Migration in Chicken Macrophages and Heterophils

**Figure 11.**
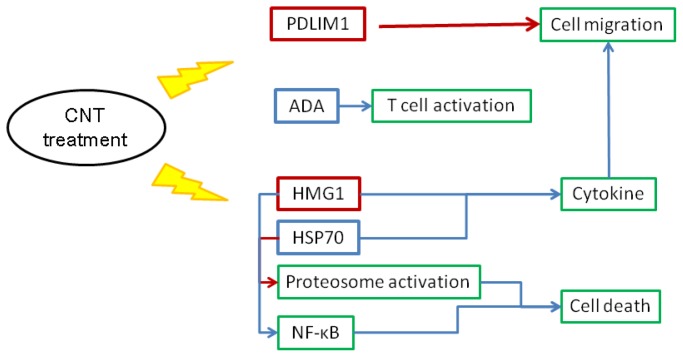
A putative mechanism of cell migration and death in chicken macrophages induced by exposure to carbon nanotubes. Blue and red frames indicate increased and decreased protein expression after carbon nanotube treatment, respectively. The green frame indicates a cellular event. The flashing lights indicate the effect induced by carbon nanotube treatment.

**Figure 12.**
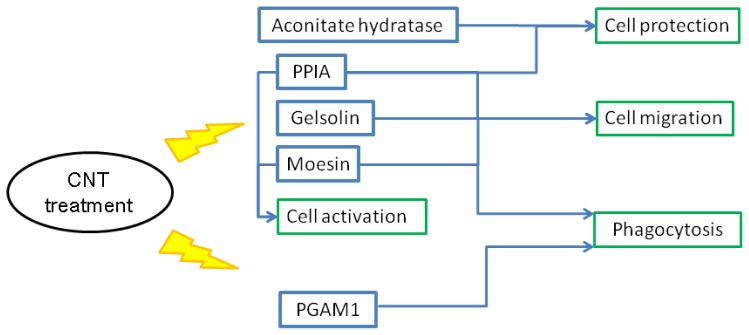
A putative mechanism of cell migration and death in chicken heterophils induced by exposure to carbon nanotubes. Blue frames indicate increased and decreased protein expression after carbon nanotube treatment, respectively. The green frame indicates a cellular event. The flashing lights indicate the effect induced by carbon nanotube treatment.

Based on the results of proteomic studies, function annotation by bioinformatic analysis and functional assays, we propose a putative mechanism of CNT-induced cell death and migration in chicken macrophages (Figure 11). In macrophages, HMG1, ADA and HSP70 respond to CNT insults, which in turn results in cytokine and chemokine production. In cooperation with PDLIM1 participation, CNT exposure promotes cell migration and thus drives chemotactic effects. Additionally, HMG1 and HSP70 are involved in cell death via the NF-κB pathway and proteasome activation. In heterophils, CNT exposure promotes cell migration by altering gelsolin, PPIA, and moesin expression patterns (Figure 12). Promotion of aconitate hydratase and PPIA expression serve to protect cells from stress. The results indicate that CNT may cause cell damage. Moreover, changes in PPIA, moesin, and PGAM1 expression related to CNT exposure can alter immune functions such as phagocytosis and cell activation.

## 3. Experimental Section

### 3.1. Source of Carbon Nanotubes

This study used multi-walled CNT with a diameter of 10–30 nm, and 2–10 μm in length. The characteristics of CNT used in this study, including its size, shape, and purity, have been examined by scanning electron microscope and thermogravimetric analysis [50]. The CNT we used was first suspended in RPMI-1640 medium (Sigma, St. Louis, MO, USA) and then sonicated for 15 min to prevent aggregation.

### 3.2. Isolation and Culture of Chicken Peripheral Blood Macrophages and Heterophils

Whole blood was collected from healthy, 30-week-old hens with heparin (Sigma) as an anticoagulant. Six hens were used in each of the three batches of experiments. Chicken macrophages and heterophils were separated by centrifugation at 1000× *g* for 30 min through a discontinuous density gradient of Percol (Sigma). Collected cells were washed twice with RPMI-1640 medium, and resuspended in Hank’s balanced salt solution (Sigma) containing 0.1% bovine serum albumin (Sigma). After culturing in RPMI-1640 medium containing CNT (0, 1, 10, and 100 μg/mL) and 10% fetal bovine serum for 6 h at 37 °C, cells were concentrated and pelleted for collection.

### 3.3. Cell Viability Assay

Cells were washed with PBS (Invitrogen, Grand Island, NY, USA), centrifuged twice to remove CNT and impurities, and then resuspended in PBS. Aliquots of cells were stained with Trypan blue (Sigma) for 5 min and cell viability was represented by a ratio of live cells to all cells.

### 3.4. Sample Preparation for Protein Analysis

For proteomic analysis, cells were washed with PBS and lysed in homogenized buffer [0.3 M sucrose (Merck, Darmstadt, Germany), 0.5 M Tris (AMRESCO, Solon, OH, USA)/HCl (Merck), and 1.67 mM Pefabloc SC PLUS (Merck)]. After centrifugation at 12,000× *g* for 5 min, the supernatant was collected and ultracentrifugated at 30,000× *g* for 1 h at 4 °C to remove CNT and impurities. Collected supernatants were then dialyzed against 100 mM ammonium bicarbonate (Sigma) for 10 h at 4 °C. Protein concentration was determined by the modified Bradford assay using BSA as the standard [51]. A total of 400 μg soluble proteins were lyophilized and solubilized in 175 μL lysis buffer [9.5 M urea (Bio-Rad Laboratories, Hercules, CA, USA), 2% NP-40 (USB Corporation, Cleveland, OH, USA), 2% *v*/*v* Ampholyte 3–10 (Serva Electroresis GmbH, Heidelberg, Germany), and 65 mM dithiothreitol (USB Corporation)] for protein analysis.

### 3.5. Analysis of Protein by Two-Dimensional Gel Electrophoresis (2-DE)

The 2-DE was performed following the procedure developed by Görg *et al.* [52] with some modifications [53]. Briefly, approximately 400 μg of total proteins in 175 μL lysis buffer were mixed well with an equal volume of rehydration buffer [8 M urea (Sigma), 2% CHAPS (Sigma), and 0.5% Pharmalyte 3–10 (GE Healthcare, Uppsala, Sweden)], and then subjected to isoelectric focusing (IEF) using immobilized pH gradient 18-cm pH 3–10 strips on Ettan *IPGphor 3* (GE Healthcare) at 20 °C. The strips were rehydrated at 30 V for 12 h and further focused for 64,000 volt-h. The strips were first equilibrated in a solution [50 mM Tris-HCl (pH 8.8), 6 M urea, 30% glycerol, 2% SDS (GE Healthcare), and 0.002% bromophenol blue (USB Corporation)] containing 100 mM dithiothreitol for 20 min and then further equilibrated in 150 mM iodoacetamide (Sigma) for 20 min. After equilibration, proteins were subjected to molecular weight separation by 12.5% SDS-PAGE using a *Daltsix* Vertical electrophoresis system (GE Healthcare). The separation was run at 15 °C with a condition of 2.5 watts per gel for 25 min followed by 9 watts per gel, until the dye front reached the bottom of the gel (typically 7–7.5 h). The molecular weight standards were obtained from Fermentas (#SM0661 Unstained Protein Ladder, Vilnius, Lithuania) containing synthesized peptides, with molecular weight ranging from 10 to 200 kDa.

### 3.6. Staining and Imaging of the 2-DE Gels

After electrophoresis, gels were stained with colloidal Coomassie blue (Serva Electrophoresis GmbH, Germany) for at least 14 h [54]. Following staining, the gels were neutralized with 0.1 M Tris/phosphoric acid (pH 6.5) for 1–3 min, and destained with 25% methanol (Merck). After destaining, gels were scanned (Image Scanner III, Lab Scan 6.0, GE Healthcare) and saved the images in TIFF format for further analysis.

### 3.7. Analysis of the Variation in Protein Expression

Protein spots on the 2-DE gels were detected and analyzed using the Melanie 7 software package (GeneBio, Geneva, Switzerland). To present variations of protein expression, spots on all 2-DE gels were quantified. The relative volume of each spot to the total volume of all spots on each gel (RVol) was generated in order to correct for differences in gel staining [55]. The Rvol was used to present the expression level of the protein spots. Spots showing significant difference were excised from the gels for identification.

### 3.8. Protein Identification by Matrix Assisted Laser Desorption/Ionization Time-of-Flight Mass Spectrometry (MALDI-TOF MS) and MALDI-TOF/TOF MS

In-gel trypsin digestion was performed according to the procedure developed by Havlis *et al.* [56] with minor modifications [53]. Gel spots were washed twice with double distilled water followed by 50% acetonitrile (ACN; Merck) in 50 mM ammonium bicarbonate and pure ACN. The gel spots were dried in a SpeedVac evaporator then subjected to in-gel digestion. For in-gel digestion, gel plugs were re-swollen with 20 ng/μL trypsin (Promega, Madison, WI, USA) in 25 mM ammonium bicarbonate at 4 °C for 30 min. Digestion was allowed to proceed for 1 h at 56 °C. After digestion, the peptides were recovered by 97.5% ACN and 2.5% trifluoracetic acid (TFA; Riedel-deHaen AG, Seelze, Germany).

MALDI-TOF MS analysis followed our previous protocols [53]. The peptides were spotted directly onto a 600 μm/384 well AnchorChip sample target (Bruker Daltonics, Bremen, Germany), and an equal volume of 1 mg/mL solution of alpha-cyano-hydroxycinnamic acid (Bruker Daltonics) in 0.1% TFA/50% ACN was added to them. The MALDI mass spectra and MALDI-TOF/TOF spectra were obtained using a Bruker *autoflex III* TOF/TOF mass spectrometer equipped with a 384 sample Scout source and delayed extraction ion source (Bruker Daltonics). An external peptide calibration standard containing Angiotensin II, Angiotensin I, Substance P, Bombesin, ACTH clip 1–17, ACTH clip 18–39 and Somatostatin 28 (Bruker Daltonics) was used to calibrate the instrument.

Data were analyzed by FlexAnalysis software (Bruker Daltonics), and the peak lists were searched against a comprehensive non-redundant protein sequence database (NCBInr 20130712 version with 30,642,817 sequences; 10,542,876,333 residues) employing the Mascot program [57], with search conditions of taxonomy of all entries, fixed modification of carbamidomethyl modification, random modification of oxidation, and mass accuracy between 50 and 100 ppm. The search conditions for MALDI-TOF/TOF spectra followed that of MALDI-TOF, except that the tolerance was set at 0.5 dalton. Positive identification was achieved with the mass accuracy and modifications set, when the score matched the protein or mixture of proteins in the database with significant probability.

### 3.9. Bioinformatic Analysis of Identified Proteins

The identified proteins were analyzed for gene ontology annotation with tools in DAVID bioinformatics, using information on the putative function of the protein found in the UniProt [58] and the Gene Ontology (GO) databases [59].

### 3.10. Induction of IL-1β

Approximately 1 × 10^7^ heterophils or macrophages were cultured in RPMI-1640 medium in the presence or absence of various levels of CNT for 4 h at 37 °C. After suspension, cells were pelleted by centrifugation at 400× *g* for 10 min and medium was collected and further concentrated (approximately 15×) using a centrifugal concentrator with a molecular weight cut-off of 5 kDa (Millpore, Billerica, MA, USA). The concentrated medium was used for total protein extraction. The IL-1β levels in the collected medium were determined by Western blot procedures.

### 3.11. Western Blot Analysis

For Western blot analysis, equal amounts of total proteins were loaded onto 10% (macrophages) and 12.5% (heterophils) SDS-PAGE, after washing three times with transfer buffer [0.5 M Tris, 0.5 M Boric acid (AMRESCO), 0.01 M EDTA (AMRESCO), pH 8.3] and being electrophoretically transferred onto the nitrocellulose membrane (Hybond-C extra; GE Healthcare) with TE77 PWR Semi-Dry Transfer Units (GE Healthcare). After blocking with Tris-buffered saline [TTBS; 20 mM Tris-HCl, pH 7.4, 500 mM NaCl (AMRESCO), 0.05% Tween 20 (USB Corporation)] containing 3% gelatin (Merck), the membranes were then incubated overnight at 4 °C with specific primary antibodies. Antibodies against HMG1, cyclophilin, α-tubulin, and HSP70 were obtained from USCNK (Houston, TX, USA), NOVUS (Littleton, CO, USA), Sigma, and Enzo Life Science (Farmingdale, NY, USA), respectively. A rabbit anti-chicken IL-1β primary antibody (Abcam, Cambridge, UK) was used to detect IL-1β levels. Membranes were washed three times with TTBS, and incubated with alkaline peroxidase-conjugated secondary antibodies (anti-mouse and anti-rabbit; Millipore, Billerica, MA, USA) for 1 h. After washing six times with TTBS, the blots were developed using enhanced chemiluminescence (Millipore), and scanned by PERFECTION 4990 photo (EPSON Taiwan Technology & Trading Ltd., Taipei, Taiwan). The protein bands were detected and analyzed with Total Lab^®^ v1.11 (UltraLum, Claremont, CA, USA).

### 3.12. Cell Migration Assay

The cell migration assay was performed according to procedures developed by Jang *et al.* [60] with some modifications. A total of 800 μL RPMI-1640 medium containing 10% BSA was loaded in a 24-well plate (Corning Inc., Corning, NY, USA). Approximately, 150 μL RPMI-1640 medium with CNT treated cells (2 × 10^5^) were loaded into the cell culture inserts (5 μm, Millipore). The inserts were put on the 24-well plate and incubated at 37 °C for 40 min. After wiping the residual cells on the upside, the migrated cells were fixed by methanol for 30 min, stained by Wright-Giemsa stain (Chroma Gesellschaft, Schmid GmbH & Co., Münster, Germany) for 15 min, and finally developed with PBS pH 7.2 for 1 min. The number of migrated cells was enumerated by light microscopy.

### 3.13. Statistical Analysis

Data from triplicate samples of the three batches were subjected to ANOVA analysis by using the general linear model procedure of Statistical Analysis System software [61]. The comparison of means among treatment was performed by the least-squares means method in the software. The difference was considered significant at *p* < 0.05.

## 4. Conclusions

In conclusion, proteomic analyses in this study showed 12 proteins expressed differentially in response to CNT exposure in chicken macrophages, and 15 in heterophils. Functional annotation through gene ontology analysis suggested that these proteins are related to cell mobility, cytokine production, and cell death. The annotated functions were further validated through cell migration, viability, and IL-1β secretion assays. Exposure to CNT differentially affects protein expression leading to activation of macrophages and heterophils, including altered cytoskeleton remodeling, cell migration, and cytokine production, and thereby mediates tissue immune responses.

## Supplementary Information



## Figures and Tables

**Table 1.** Differentially expressed protein spots in carbon nanotube-treated chicken macrophages. #

Spot No. *	Protein Identity	Concentration of Carbon Nanotube			

		Control (0 μg/mL)	1 μg/mL	10 μg/mL	100 μg/mL
27	Protein MRP-126	1.310 ± 0.128 b	1.464 ± 0.082 a,b	1.838 ± 0.363 a	1.182 ± 0.042 b
30	similar to D4-GDP-dissociation inhibitor	0.290 ± 0.058 a,b	0.332 ± 0.031 a	0.269 ± 0.013 a,b	0.196 ± 0.049 b
57	similar to hepatoma-derived growth factor (high-mobility group protein 1-like)	0.193 ± 0.033 b	0.191 ± 0.027 b	0.298 ± 0.030 a	0.218 ± 0.027 a,b
69	high mobility group protein HMG1	0.660 ± 0.061 a	0.622 ± 0.015 a	0.530 ± 0.113 a,b	0.351 ± 0.019 b
94	similar to Pdlim1 protein isoform 1	0.378 ± 0.051 a	0.410 ± 0.049 a	0.407 ± 0.022 a	0.161 ± 0.056 b
107	adenosine deaminase	0.168 ± 0.011 b	0.161 ± 0.021 b	0.185 ± 0.009 b	0.254 ± 0.020 a
108	actin, cytoplasmic type 5	2.256 ± 0.669 b,c	1.848 ± 0.534 c	3.833 ± 0.837 a,b	4.067 ± 0.269 a
109	beta-actin	2.186 ± 0.241 a,b	2.958 ± 0.316 a	1.806 ± 0.248 b	2.337 ± 0.197 a,b
140	heat shock protein 70	0.337 ± 0.041 b	0.330 ± 0.027 b	0.310 ± 0.047 b	0.488 ± 0.056 a
153	phosphoglycerate kinase	0.975 ± 0.117 a	0.733 ± 0.093 a,b	0.484 ± 0.116 b	0.747 ± 0.152 a,b
156	NS	3.204 ± 0.373 a,b	2.855 ± 0.518 a,b	1.852 ± 0.207 b	4.010 ± 1.104 a
199	NS	0.716 ± 0.082 a	0.389 ± 0.069 b	0.505 ± 0.134 a,b	0.389 ± 0.061 b

# The protein expression was represented as the ratio of the volume of a spot to the overall volume of the quantified spots (mean ± SE of three batches) generated by the Malaline 7 software and used to present the expression level of a protein spot;

* The spot numbers refer to the numbers labeled in Figure 1;

a–c The means in the same row with different superscripts differ significantly (*p* < 0.05);

NS: No significant match in the database.

**Table 2.** Differentially expressed protein spots in carbon nanotube-treated chicken heterophils. #

Spot No. *	Protein Identity	Concentration of Carbon Nanotube			

		Control (0 μg/mL)	1 μg/mL	10 μg/mL	100 μg/mL
60	gelsolin precursor	0.131 ± 0.018 b	0.168 ± 0.029 a,b	0.201 ± 0.037 a,b	0.227 ± 0.030 a
78	peptidylprolyl isomerase A (cyclophilin A)	0.723 ± 0.191 b	0.754 ± 0.090 b	0.885 ± 0.191 a,b	1.275 ± 0.057 a
80	recombination activating protein 1	0.713 ± 0.043 b	1.191 ± 0.222 a	1.359 ± 0.163 a	1.660 ± 0.213 a
87	NS	0.103 ± 0.002 b	0.212 ± 0.028 a	0.161 ± 0.033 a,b	0.186 ± 0.042 a,b
118	aconitate hydratase, mitochondrial	0.063 ± 0.016 b	0.109 ± 0.023 a,b	0.131 ± 0.026 a	0.093 ± 0.010 a,b
123	moesin-like	0.221 ± 0.008 a,b	0.222 ± 0.009 a,b	0.286 ± 0.058 a	0.175 ± 0.011 b
143	moesin-like	0.151 ± 0.039 b	0.178 ± 0.034 a,b	0.197 ± 0.028 a,b	0.272 ± 0.012 a
156	cell division control protein 42 homolog precursor	0.255 ± 0.012 a,b	0.309 ± 0.040 a	0.186 ± 0.017 b	0.225 ± 0.018 b
161	phosphoglycerate mutase 1	0.161 ± 0.022 b	0.203 ± 0.006 a,b	0.169 ± 0.013 b	0.250 ± 0.015 a
164	glyceraldehyde-3-phosphate dehydrogenase	0.737 ± 0.143 b	1.338 ± 0.144 a	0.777 ± 0.219 b	0.411 ± 0.077 b
172	peptidyl-prolyl cis-trans isomerase FKBP4	0.077 ± 0.007 b	0.138 ± 0.028 a	0.103 ± 0.003 a,b	0.084 ± 0.015 b
188	beta-actin	0.246 ±0.045 a,b	0.254 ± 0.068 a,b	0.356 ± 0.092 a	0.150 ± 0.015 b
195	hypothetical protein RCJMB04_1a14	0.110 ± 0.023 a	0.063 ± 0.005 b	0.073 ± 0.007 a,b	0.063 ± 0.005 b
219	phosphoglycerate kinase	0.905 ± 0.050 a	0.563 ± 0.131 b	0.833 ± 0.064 a	0.525 ± 0.092 b
220	similar to transketolase	0.195 ± 0.013 a	0.171 ± 0.028 a	0.149 ± 0.011 a,b	0.112 ± 0.011 b

# The protein expression was represented as the ratio of the volume of a spot to the overall volume of the quantified spots (mean ± SE of three batches) generated by the Malaline 7 software and used to present the expression level of a protein spot;

* The spot numbers refer to the numbers labeled in Figure 2;

a,b The means in the same row with different superscripts differ significantly (*p* < 0.05);

NS: No significant match in the database.
