# Supplementary material for: Functional Annotation of Proteomic Data from Chicken Heterophils and Macrophages Induced by Carbon Nanotube Exposure
Source: Int J Mol Sci. 2014 May 12;15(5):8372–92. doi: 10.3390/ijms15058372 (PMC4057737; doi:10.3390/ijms15058372)
Supplement: Supplementary file 1 [file ijms-15-08372-s001.pdf]

Supplementary Information

Table S1. List of differentially expressed chicken macrophages proteins after treated with carbon nanotube identified by peptide mass fingerprinting.

Spot No. [a]	Protein Identity	GeneBank Accession	Source Species	MALDI-MS/PMF [b]	Score	Theoretical Mr/pI	MALDI-TOF/TOF [c]	Functional Classification	Subcellular Location
M27	Protein MRP-126	gi|126659	*Gallus gallus*	NS	-	14,172/6.44	1 (84) 84	Calcium ion binding (ion binding)	Cytoplasm
M30	similar to D4-GDP-dissociation inhibitor	gi|50728568	*Gallus gallus*	NS	-	22,929/5.08	4 (93, 63, 12) 167	Rho GDP-dissociation inhibitor activity (enzyme regulator activity)	Cytoplasm
M57	similar to hepatoma-derived growth factor (high-mobility group protein 1-like)	gi|118107483	*Gallus gallus*	9/58 (49)	97	20,684/4.80	2 (19, 66) 79	Growth factor activity (protein binding)	Cytoplasm
M69	high mobility group protein HMG1	gi|5815432	*Gallus gallus*	2/20 (21)	68	24,952/5.75	5 (69, 56, 82, 105) 310	Cytokine activity (protein binding)	Cytoplasm
M94	similar to Pdlim1 protein isoform 1	gi|118092685	*Gallus gallus*	15/66 (66)	131	36,094/7.00	1 (59) 59	Zinc ion binding (ion binding)	Nucleus
M107	adenosine deaminase	gi|57529377	*Gallus gallus*	20/62 (84)	168	41,052/5.80	3 (53, 30, 25) 109	Adenosine deaminase activity (catalytic activity)	Cytoplasm
M108	actin, cytoplasmic type 5	gi|56119084	*Gallus gallus*	25/86 (50)	131	42,157/5.30	2 (61, 130) 191	Identical protein binding (protein binding)	Cytoskeleton
M109	beta-actin	gi|63018	*Gallus gallus*	17/40 (49)	157	42,086/5.29	3 (40, 79, 108) 226	Protein binding	Cytoskeleton
M140	heat shock protein 70	gi|30962014	*Gallus gallus*	35/130 (36)	90	70,098/5.50	3 (17, 50, 75) 142	Protein binding	Cell surface
M153	phosphoglycerate kinase	gi|45384486	*Gallus gallus*	18/104 (47)	78	45,087/9.20	2 (139, 60) 199	Phosphoglycerate kinase activity (catalytic activity)	Cytoplasm
M156	NS	-	-	-	-	-	-	-	-
M199	NS	-	-	-	-	-	-	-	-

[a] The spot numbers refers to the numbers labled on Figure 2; [b] The column refers to the results of the MALDI-MS analysis, *i.e.*, the number of assigned and percent sequence coverage (in brackets); [c] The column refers to the results of the MALDI-TOF/TOF analysis, *i.e.*, to the summary score and Mowse scores (in brackets) of assigned peptides; NS: no significant match in the database.

Table S2. List of differentially expressed chicken heterophils proteins after treated with carbon nanotube identified by peptide mass fingerprinting.

Spot No. [a]	Protein Identity	GeneBank Accession	Source Species	MALDI-MS/ PMF [b]	Score	Theoretical Mr/pI	MALDI-TOF/TOF [c]	Functional Classification	Subcellular Location
H60	gelsolin precursor	gi|45384386	*Gallus gallus*	10/58 (36)	86/74	86,120/5.90	2 (20, 29) 49/45	Actin binding (protein binding)	Cytoplasm
H78	peptidylprolyl isomerase A (cyclophilin A)	gi|261490820	*Gallus gallus*	11/72 (50)	74/74	18,084/9.50	2 (56, 68) 125/43	Peptide binding (binding)	Cytoplasm
H80	recombination activating protein 1	gi|241994242	*Ochthoeca cinnamomeiventris*	18/76 (26)	85/74	112,026/8.83	NS	Histone binding (protein binding)	Nucleus
H87	Hypothetical protein	gi|118102987	*Gallus gallus*	NS	79/74	62,606/5.00	1 (102) 102/45	NS	NS
H118	aconitate hydratase, mitochondrial	gi|45383738	*Gallus gallus*	24/75 (39)	153/74	86,535/8.89	2 (23, 23) 46/45	Iron ion binding (ion binding)	Mitochondrion
H123	moesin-like	gi|326924179	*Meleagris gallopavo*	29/81 (44)	137/74	68,220/5.90	3 (39, 42, 21) 103/45	Cell adhesion molecule binding (protein binding)	Cytoskeleton
H143	moesin-like	gi|326924179	*Meleagris gallopavo*	19/67 (27)	98/74	68,220/5.90	-	Cell adhesion molecule binding (protein binding)	Cytoskeleton
H156	cell division control protein 42 homolog precursor	gi|45384262	*Gallus gallus*	NS	NS	21,601/6.15	2 (35, 64) 99/45	GTP binding (binding)	Cytoplasm
H161	phosphoglycerate mutase 1	gi|71895985	*Gallus gallus*	9/39 (50)	82/74	29,051/7.80	1 (38) 38/44	Phosphoglycerate mutase activity (catalytic activity)	Cytoplasm

Table 2. *Cont.*

Spot No. [a]	Protein Identity	GeneBank Accession	Source Species	MALDI-MS/ PMF [b]	Score	Theoretical Mr/pI	MALDI-TOF/TOF [c]	Functional Classification	Subcellular Location
H164	glyceraldehyde-3-phosphate dehydrogenase	gi|46048961	*Gallus gallus*	16/74 (20)	82/74	35,909/9.40	2 (91, 60) 151/45	Glyceraldehyde-3-phosphate dehydrogenase (NAD+) (phosphorylating) activity (catalytic activity)	Cytoplasm
H172	peptidyl-prolyl cis-trans isomerase FKBP4	gi|57525441	*Gallus gallus*	10/18 (43)	131/74	50,742/5.40	2 (54, 2) 56/44	Heat shock protein binding (protein binding)	Nucleus
H188	beta-actin	gi|63018	*Gallus gallus*	NS	NS	42,086/5.29	3 (23, 55, 11) 88/44	Protein binding	Cytoskeleton
H195	hypothetical protein RCJMB04_1a14	gi|53126140	*Gallus gallus*	16/54 (25)	101/74	86,394/8.90	NS	Iron ion binding (ion binding)	Mitochondrion
H219	phosphoglycerate kinase	gi|45384486	*Gallus gallus*	NS	108/74	45,094/8.31	2 (40, 36) 76/44	Phosphoglycerate kinase activity (catalytic activity)	Cytoplasm
H220	similar to transketolase	gi|118096822	*Gallus gallus*	20/72 (41)	139/74	69,152/7.90	2 (67, 37) 103/45	Magnesium ion binding (ion binding)	Cytoplasm

[a] The spot numbers refers to the numbers labled on Figure 4; [b] The column refers to the results of the MALDI-MS analysis, *i.e.*, the number of assigned and percent sequence coverage (in brackets); [c] The column refers to the results of the MALDI-TOF/TOF analysis, *i.e.*, to the summary score and Mowse scores (in brackets) of assigned peptides; NS: no significant match in the database.

© 2014 by the authors; licensee MDPI, Basel, Switzerland. This article is an open access article distributed under the terms and conditions of the Creative Commons Attribution license (http://creativecommons.org/licenses/by/3.0/).
